# Inhibition of Nitric Oxide Production in Activated Macrophages Caused by *Toxoplasma gondii* Infection Occurs by Distinct Mechanisms in Different Mouse Macrophage Cell Lines

**DOI:** 10.3389/fmicb.2018.01936

**Published:** 2018-08-20

**Authors:** Gabriel R. de Abreu Cabral, Zi T. Wang, L. D. Sibley, Renato A. DaMatta

**Affiliations:** ^1^Department of Molecular Microbiology, Washington University School of Medicine, St. Louis, MO, United States; ^2^Laboratório de Biologia Celular e Tecidual, Centro de Biociências e Biotecnologia, Universidade Estadual do Norte Fluminense, Campos dos Goytacazes, Brazil

**Keywords:** *Toxoplasma gondii*, virulence factors, macrophages, inducible nitric oxide synthase, nitric oxide

## Abstract

*Toxoplasma gondii*, the causative agent of toxoplasmosis, is a widespread intracellular parasite able to infect virtually any nucleated cell. *T. gondii* infection of activated macrophages inhibits nitric oxide (NO) production; however, parasite effectors responsible for this block have not been defined. Macrophage populations are extremely heterogeneous, responding differently to stimuli and to parasite infection. Here we evaluated the inhibition of NO production caused by *T. gondii* infection of J774-A1 and RAW 264.7 macrophages and assessed the role of several known parasite virulence factors in this phenotype. Infection of activated macrophages from both macrophage lines reduced NO production, however, the mechanism of this decrease was different. Consistent with previous reports, infected J774-A1 macrophages had reduced iNOS expression and lower number of iNOS positive cells. In contrast, *T. gondii* infection of RAW 264.7 macrophages did not alter iNOS expression or the number of iNOS positive cells, and yet it led to lower levels of NO production. Deletion of a number of previously defined virulence factors including ROP kinases that disrupt innate immune factors, TgIST which blocks STAT1 activation, as well as the secretory trafficking proteins ASP5 and MYR1, did not alter the phenotype of decreased NO production. Taken together our findings indicate that *T. gondii* infection inhibits NO production of activated macrophages by different mechanisms that involve reduction of iNOS expression vs. iNOS impairment, and suggest that a novel parasite effector is involved in modulating this important host defense pathway.

## Introduction

Toxoplasmosis is a worldwide disease affecting about one-third of the human population (Tenter et al., 2000). *Toxoplasma gondii*, the causative agent of toxoplasmosis, is an obligate intracellular parasite that infects distinct host cells (Tenter et al., 2000). Macrophages are key players of the host immune system and are able to control *T. gondii* replication following activation by interferon gamma and a second signal provide by LPS or TNF-α (Adams et al., 1990; Sibley et al., 1991). One of the main components of antimicrobial activities of activated macrophages is the production of NO through the induction of iNOS (MacMicking et al., 1997). Generation of NO has been implicated in control of chronic toxoplasmosis (Chao et al., 1993; Khan et al., 1997; Scharton-Kersten et al., 1997; Roberts et al., 2000). However, *T. gondii* coevolved with its host and evasion mechanisms have emerged to thwart many of the effectors of activated macrophages. Included among these virulence factors are proteins released from rhoptries that block innate immunity (Hunter and Sibley, 2012) and dense granules that alter host cell transcription (Hakimi et al., 2017).

During host cell invasion, *T. gondii* secretes contents from specialized secretory organelles including rhoptries and dense granules that have a central role in parasitophorous vacuole formation and host immunity subversion (Carruthers and Sibley, 1997; Bougdour et al., 2013; Braun et al., 2013; Etheridge et al., 2014; Olias et al., 2016). For example, the ROP5-ROP17-ROP18 complex, which is secreted from rhoptries, blocks the assembly and function of vacuolar-targeted IRGs (Saeij et al., 2006; Taylor et al., 2006; Behnke et al., 2011; Reese et al., 2011; Etheridge et al., 2014). Recruitment of IRGs to the vacuole normally results in its destruction and death of the parasite (Zhao et al., 2009; Khaminets et al., 2010), but ROPs of virulent strains of the parasite are able to phosphorylate key IRG proteins, inhibiting their activity and assembly, protecting *T. gondii* (Fentress et al., 2010; Steinfeldt et al., 2010). Proteins from GRA are another important class of virulence factors secreted by *T. gondii* during and after host cell invasion that perform major roles in parasite survival and replication (Mercier et al., 2002). For example, GRA16 down-modulates host p53 expression changing the cell cycle (Bougdour et al., 2013), while GRA24 causes host p38α activation, leading to a strong proinflammatory response (Braun et al., 2013). Recently, another important *T. gondii* virulence factor known as inhibitor of STAT1-dependent transcription (IST) has been described (Gay et al., 2016; Olias et al., 2016). IST translocates to the host nucleus where it recruits a repressive complex of STAT1 promoters, blocking the IFN-γ dependent transcription, avoiding host cell activation (Olias et al., 2016).

Classically activated macrophages produce NO that control *T. gondii* replication (Adams et al., 1990; Bohne et al., 1994; Khan et al., 1997). NO is an important microbicidal molecule that is produced by iNOS (Stuehr et al., 1991; Xie et al., 1994; Lowenstein and Padalko, 2004). It is well known that *T. gondii* evades the cytotoxic effects of NO by inhibiting NO production of activated mice peritoneal macrophages (Dobbin et al., 2002; Seabra et al., 2002, 2004; Luder et al., 2003). Furthermore, in activated J774-A1 macrophages, infection causes iNOS degradation by the proteasome (Padrao Jda et al., 2014). Although TGF-β 1 is involved in the inhibition of NO production in infected activated macrophages (Seabra et al., 2004), the parasite effector responsible for iNOS degradation and NO inhibition still remains elusive. In addition, most of these studies have been done *in vitro* with a single cell type and without comparison of other macrophage cell lines.

Macrophages are an extremely heterogeneous cell population with many subpopulations that behave differently (Geissmann et al., 2010). Thus, *T. gondii* has to deal with many distinct macrophage subpopulations during host infection. To better understand how *T. gondii* copes with NO production of distinct activated macrophages lines, production of this microbicidal molecule and expression of iNOS were compared in two macrophage cell lines after infection. In addition, several previously described virulence factors were also analyzed as possible effectors responsible for NO inhibition of infected activated macrophages. This study reveals intrinsic differences between both macrophage cell lines in activation patterns and mechanisms by which *T. gondii* infection disrupted NO production. Furthermore, previously identified virulence effectors that were tested here did not alter the NO inhibition phenotype detected in both macrophages cell lines, indicating that a novel effector is responsible for the inhibition of this important host microbicidal molecule.

## Materials and Methods

### Biosecurity and Institutional Safety Procedures

This study was carried out in accordance with the NIH standard biosecurity and institutional safety procedures of Washington University School of Medicine.

### *Toxoplasma gondii* and Cell Culture

Knockout parasites used in this work were previously generated as reported: RHΔku80 (Fox et al., 2009); Δrop5 (Behnke et al., 2011); Δrop17, Δrop17/18, Δrop18 (Etheridge et al., 2014); ΔTgIST (Olias et al., 2016); (Δasp5) (Curt-Varesano et al., 2016); (Δmyr1) (Franco et al., 2016). Wild type *T. gondii* tachyzoites, deficient in Ku80 (RHΔku80) and knockout parasites, all of the RH strain, were maintained by serial passage in Human Foreskin Fibroblast (HFF) monolayers. Parasites were released from HFF using a cell scraper (TPP, Switzerland). The cell suspension was passed three times in a 10 ml syringe (BD, United States) with a 22-gauge needle (CML Supply, United States) and it was filtered on a 3 μm Whatman Nuclepore membrane (GE Healthcare Life Sciences, United States).

Human Foreskin Fibroblast cells were obtained from the Boothroyd laboratory at Stanford University. HFF cells and the mouse macrophage cell lines RAW 264.7 (ATCC TIB-71, United States, from BALB/c mice ascites after Abelson murine leukemia virus inoculation) and J774-A1 (ATCC TIB-67, United States, from BALB/c mice ascites after reticulum cell sarcoma inoculation) were cultivated in Dulbecco’s modified Eagle’s medium (DMEM - Invitrogen, United States) supplemented with 10% HyClone Fetal Bovine Serum (FBS - GE Healthcare Life Sciences, United States), 10 mM glutamine (Thermo Fisher Scientific, United States) and 10 μg/ml gentamicin (Gibco, United States) in an incubator (Thermo Fisher Scientific, United States) at 37°C in 5% CO_2_ atmosphere. Cultures were negative for *Mycoplasma* spp. contamination with the e-Myco plus PCR detection kit (Boca Scientific, United States).

### Generation of Δrop16 Knockout Parasites

The knockout plasmid construct for *ROP16* was created using the three-fragment Gateway™ recombination system (Invitrogen, United States), joining two constructs homologous to the 5′ and 3′ flanks of *ROP16* with a central HXGPRT expression cassette, as described previously (Etheridge et al., 2014). This plasmid was transfected into RHΔhxgprtΔku80 parasites, and resistant pools were expanded under treatment with mycophenolic acid (15 μg/ml) acid and xanthine (50 μg/ml). After PCR (Applied Biosystems, United States) confirmation of construct integration in the pool, parasites were subcloned by limiting dilution in 96-well plates (TPP, Switzerland) containing confluent monolayers of HFF cells. Clones were identified by visual confirmation of single plaques, screened by PCR to confirm replacement of the coding region of ROP16 with the HXGPRT cassette, expanded by growth on HFF monolayers, and cryo-preserved.

### Macrophage Activation and Infection

The RAW 264.7 and J774-A1 cells were seeded at the density of 5 × 10^4^ cells per well in 96-well plates, activated with 200 U/ml of recombinant mouse IFN-γ (R&D Systems, United States) and 0.2 μg/ml of LPS from *Escherichia coli* 0111:B4 (Sigma-Aldrich, United States) for 24 h prior of the *T. gondii* infection. After 24 h of activation, cells were washed twice with sterile PBS, DMEM supplemented with 3% FBS was added, cells were infected with a 5 *T. gondii* per macrophage (5:1) ratio and incubated at 37°C for 2 h. The *T. gondii*-macrophage ratio used was determined by previous experiments examining NO production and number of adhered macrophages after 24 h infection. After infection, cells were washed twice in sterile PBS, and DMEM supplemented with 10% FBS with IFN-γ and LPS or these activators and L-arginine (Sigma-Aldrich, United States) at different concentration, was added. Supernatants were collected at 2, 4, 6, and 24 h after infection for the nitrite assay.

### Nitrite Oxide (NO) Production

The NO produced by macrophages was assayed by the Griess reagent (Green et al., 1982). Briefly, 50 μl of cell supernatant from each well were collected and distributed in 96-well plates and 50 μl of Griess reagent (1:1, 0.1% N-(1-Naphthyl) ethylenediamine dihydrochloride (Sigma-Aldrich, United States) in distilled water and 1% sulfanilamide (Sigma-Aldrich, United States) in 5% phosphoric acid (Sigma-Aldrich, United States) were added. Plates were incubated at room temperature and nitrite was read in a plate reader (BioTek, United States) at 540 nm. The nitrite value was calculated from a calibrated standard curve using sodium nitrite ranging from 0 to 100 μM.

### In-Cell-ELISA

Infection of parental and knockout parasites into RAW 264.7 and J774-A1 macrophages was evaluated after 2 h of challenge in cells seeded in 96-well plates, activated and infected as described. After infection, cells were fixed and permeabilized for 30 min in PBS containing 4% formaldehyde (Polysciences, Inc., United States), 0.05% Triton X-100 (Fisher BioReagents, United States), washed with PBS and blocked for 30 min with 5% FBS and Normal Goat Serum (Sigma-Aldrich, United States) in PBS (FBS-NGS). Cells were incubated for 1 h with anti-RH (SAG1) rabbit antibody diluted 1:2000 in FBS-NGS, washed twice and incubated for 1 h with anti-rabbit HRP conjugated antibody (Life Technology, United States) diluted 1:4000 in FBS-NGS. Cells were washed three times, incubated for 15 min with 100 μl of TMB substrate (BD OptEIA, Thermo Fisher Scientific, United States), reaction stopped with 100 μl of 1M H_2_SO_4_ and the absorbance was read in a plate reader (BioTek, United States) at 450 nm. Non-infected macrophages were used as negative control.

### Immunofluorescence Assay

RAW 264.7 and J774-A1 macrophages were seeded at the density of 5 × 10^5^ cells per well over coverslips (Fisherbrand, United States) in 24-well plates (TPP, Switzerland), activated and infected as described. Cells were fixed for 30 min with PBS containing 4% formaldehyde, permeabilized for 15 min in PBS containing 0.1% Triton X-100, incubated for 30 min with PBS containing 100 mM of NH_4_Cl (Sigma-Aldrich, United States), and washed three times with PBS containing 1.5% Bovine Serum Albumin (PBS-BSA, Sigma-Aldrich, United States). Cells were incubated for 1 h with anti-iNOS mouse monoclonal antibody (sc-7271, Santa Cruz Biotechnology, United States) diluted 1:100 and anti-RH (SAG1) rabbit antibody diluted 1:2000 in PBS-BSA, washed once in PBS and twice in PBS-BSA and incubated with goat anti-mouse IgG monoclonal antibody conjugated to Alexa Fluor 488 (Thermo Fisher Scientific, United States) diluted 1:200 and goat anti-rabbit IgG monoclonal antibody conjugated to Alexa Fluor 594 (Thermo Fisher Scientific, United States) diluted 1:2000 in PBS-BSA. Coverslips containing cells were mounted with the Prolong Gold antifade reagent with DAPI (Life Technologies, United States) and visualized with a Zeiss Axioskop 2 MOT Plus epifluorescence microscope with a 63× Plan Apochromat lens (numerical aperture of 1.40; Carl Zeiss, Inc., Germany), equipped with an AxioCam MRm camera (Carl Zeiss, Inc., Germany). Images were acquired using Axiovision v4.6 (Carl Zeiss, Inc., Germany) and processed with Adobe Photoshop 6.0 (Adobe Systems Inc., United States).

### Cell Analysis

To quantify the percentage of iNOS positive and iNOS negative cells in infected or non-infected cells, RAW 264.7 and J774-A1 macrophages were seeded in black 96-well cellstar microplates with clear bottom and condensation rings (BioTek, United States), activated and infected as described. Cells were fixed with PBS containing 4% formaldehyde for 15 min, washed three times with PBS and incubated for 15 min with FBS-NGS containing 5 μg/ml of Wheat Germ Agglutinin conjugated to Alexa Fluor 633 (Thermo Fisher Scientific, United States) for total cell staining. Cells were washed twice in PBS and incubated for 1 h with anti-iNOS mouse monoclonal antibody (Santa Cruz Biotechnology, United States) diluted 1:100 in FBS-NGS and anti-RH (SAG1) rabbit antibody diluted 1:10000 in 5% FBS-NGS. Cells were washed twice with PBS and incubated with goat anti-mouse IgG conjugated to Alexa Fluor 488 (Thermo Fisher Scientific, United States) diluted 1:200 and goat anti-rabbit IgG conjugated to Alexa Fluor 594 (Thermo Fisher Scientific, United States) diluted 1:2000 in FBS-NGS. Cell analysis was performed with a Cytation 3 (BioTek, United States) multimode plate imager equipped with Gen5 software and 20× objective.

### Western Blot and Densitometry Analysis

RAW 264.7 and J774-A1 macrophages were seeded at the density of 2 × 10^6^ cells per well in 6-well plate (TPP, Switzerland), activated and infected as described. Cells were washed once in sterile PBS and lysed with 50 μl of lysing buffer containing 50 mM of Tris–HCL (Sigma-Aldrich, United States), 150 mM NaCl (Sigma-Aldrich, United States), 1% Triton X-100 (Sigma-Aldrich, United States) and protease inhibitor cocktail (Sigma-Aldrich, United States). Protein samples were frozen in liquid nitrogen three times, centrifuged (Eppendorf, Germany) at 5,000 *g*, for 3 min and protein concentration was measured using the Pierce BCA Protein Assay Kit (Thermo Fisher Scientific, United States). Samples were diluted 4:1 in 5× Laemmli buffer containing 10 mM of dithiothreitol (Sigma-Aldrich, United States), boiled for 5 min, resolved in 8% polyacrylamide gels (Bio-Rad Laboratories, Inc., United States) by SDS–PAGE, and transferred to nitrocellulose membranes Amersham Protran 0.45 NC (GE Healthcare Life Sciences, United States) for 1 h. Membranes were blocked overnight at 4°C with 5% fat-free milk in PBS 0.1% Tween 20 (Sigma-Aldrich, United States), probed for 1 h with anti-iNOS mouse monoclonal antibody (Santa Cruz Biotechnology, United States) dilute 1:1000 and rabbit anti-β-actin (Cell Signaling Technology, United States) diluted 1:1000 in 5% fat-free milk in PBS 0.1% Tween 20. Membranes were washed with PBS 0.1% Tween 20 and labeled for 1 h with IR dye-conjugated secondary antibodies (LI-COR Biosciences, United States) against mouse and rabbit dilute 1:15000 and visualized on a LiCor Odyssey Imaging System (LI-COR Biosciences, United States). Western blots were quantified using the software ImageJ and intensity values were normalized to β-actin at each time interval.

### Statistical Analysis

Statistical analyses were conducted with Prism 7 (GraphPad Software Inc., United States), and differences in the means were assessed by one-way or two-way ANOVA with Tukey’s multiple comparison post-test, or unpaired Student’s *t*-test. *P* ≤ 0.05 was the cutoff considered minimum for significance.

## Results

### *T. gondii* Inhibits NO Production of Activated Macrophages Independently of Substrate Availability and Cell Type

**FIGURE 1:**
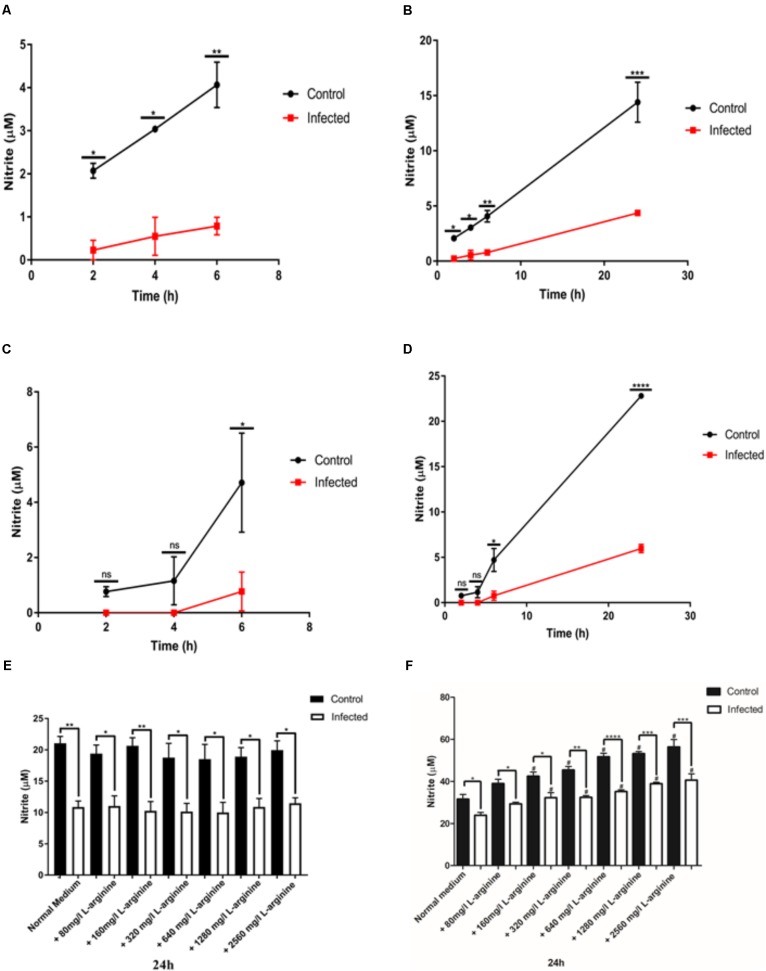
Nitric oxide (NO) production (nitrite in μM) in activated J774-A1 and RAW 264.7 cells macrophages after *T. gondii* infection. **(A)** NO production of non-infected (Control) or *T. gondii* (RH) infected J774-A1cells at 6 h and **(B)** 24 h post-infection. Mean ± SEM (*n* = 3 experiments, each with 12 replicates). **(C)** NO production of non-infected (Control) or *T. gondii* (RH) infected RAW 264.7 cells at 6 h and **(D)** 24 h post-infection. Mean ± SEM (*n* = 3 experiments, each with 12 replicates). ns (not significant), ^∗^*P* ≤ 0.05, ^∗∗^*P* ≤ 0.01, ^∗∗∗^*P* ≤ 0.001, two-way ANOVA with Tukey post-test. **(E)** NO production of non-infected (Control) or *T. gondii* (RH) infected J774-A1 **(F)** or RAW 264.7 cells for 24 h with normal medium or supplemented with different levels of L-arginine. Mean ± SEM (*n* = 3 experiments, each with 6 replicates). ^∗^*P* ≤ 0.05 and ^∗∗^*P* ≤ 0.01, ^∗∗^*P* ≤ 0.01, ^∗∗∗^*P* ≤ 0.001, ^∗∗∗∗^*P* ≤ 0.0001 one-way ANOVA with Tukey post-test, ^#^*P* ≤ 0.05 comparing the “Control” or “Infected” bar with the respective “Normal medium” bar.

To evaluate the ability of the parasite to inhibit NO production in different macrophage cell lines, activated J774-A1 or RAW 264.7 cells were infected with *T. gondii* and nitrite production was evaluated in culture supernatant at different time points. Differences in the timing of inhibition of NO production were observed between the two cell lines. In J774-A1 macrophages, inhibition of NO production occurred by 2 h post-infection (**Figure 1A**) and was sustained up to 24 h (**Figure 1B**). In contrast, inhibition of NO production in RAW 264.7 macrophages occurred only after 6 h post-infection (**Figure 1C**) and was sustained up to 24 h (**Figure 1D**).

During host cell invasion *T. gondii* secretes important virulence factors including ROP16, which activates STAT 3 and STAT 6 in macrophages resulting in upregulation of arginase 1 (ARG1) (Butcher et al., 2011). Induction of ARG1 competes with iNOS for the substrate L-arginine (Butcher et al., 2011), hence this may be a mechanism of avoiding NO production. To determine if inhibition of NO production in activated macrophages was caused by substrate depletion, we supplemented the culture medium with increasing concentrations of L-arginine and evaluated NO production after 24 h of infection. Treatment of activated J774-A1 macrophages with increasing concentrations of L-arginine did not enhance NO production in control or infected cells, nor reverted the inhibition of NO production phenotype caused by *T. gondii* infection (**Figure 1E**). In contrast, incubation of activated RAW 264.7 macrophages with increasing concentrations of L-arginine enhanced NO production in control and infected cells, and yet it did not reverse the inhibition of NO production in infected cells (**Figure 1F**).

### iNOS Expression in Infected Macrophages Is Dependent on Cell Line Type

**FIGURE 2:**
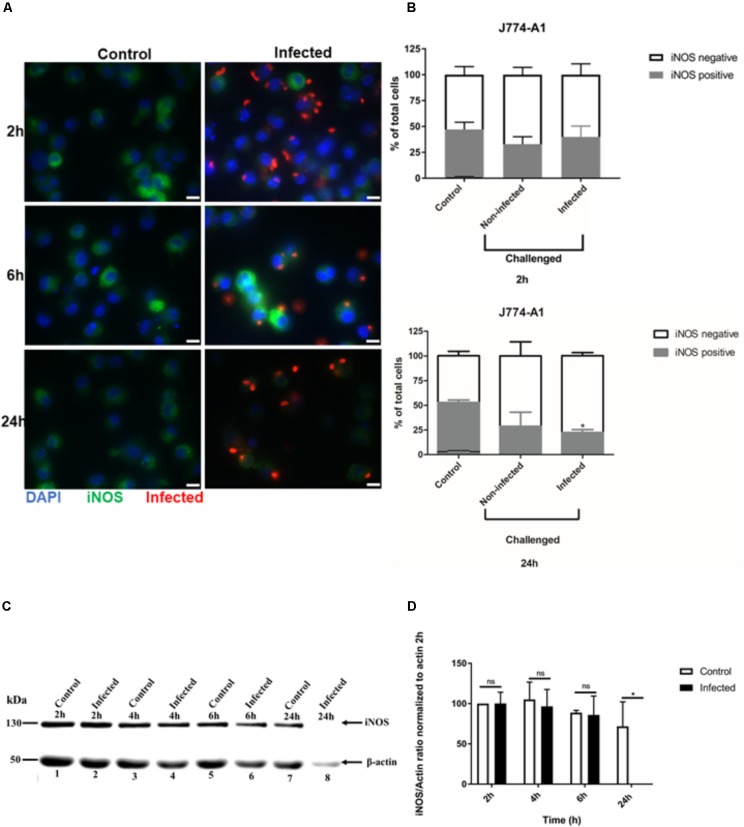
Immunofluorescence detection of iNOS in activated J774-A1 macrophages infected with *T. gondii*. **(A)** Detection of iNOS (green) in non-infected (Control) and in *T. gondii* (red) infected cells (DAPI - blue) at 2, 6, and 24 h post-infection. Scale bar = 10 μm. **(B)** Analysis of the proportion of iNOS positive or negative macrophages in non-infected (Control) and *T. gondii* infected cells at 2 and 24 h post-infection. Mean ± SEM (*n* = 4 experiments, each with 8 replicates). **(C)** Western blot detection of iNOS expression in non-infected (Control) and *T. gondii* infected (Infected) cells. β-actin was used as loading control. **(D)** Densitometry of western blots normalized to β-actin at 2 h post-infection. Mean ± SD (*n* = 3 experiments, each with 1 replicate). ^∗^*P* ≤ 0.05, two-way ANOVA with Tukey post-test, n.s (not significant).

To examine whether the inhibition of NO production in activated J774-A1 macrophage was caused by protein degradation, iNOS expression was evaluated by different methods. iNOS showed variable levels of expression in activated non-infected J774-A1 macrophages based on IFA staining (**Figure 2A**). Following infection with *T. gondii* there were more iNOS negative cells at 24 h post-infection (**Figure 2A**). This finding was confirmed by analyzing the proportion of activated cells that were positive for iNOS in non-infected cells (Control) and *T. gondii* challenged cells (Non-infected or Infected) as depicted in **Figure 2B**. Although there was a tendency to decrease the number of iNOS positive cells in infected macrophages at 2 h, this effect was significant at 24 h post-infection (**Figure 2B**). Western blot (**Figure 2C** and **Supplementary Figure 1**) and densitometry analysis (**Figure 2D**) also confirmed inhibition of iNOS expression in cells infected for 24 h. However, reduction in iNOS expression was not observed at earlier time points after infection (**Figure 2D**).

**FIGURE 3:**
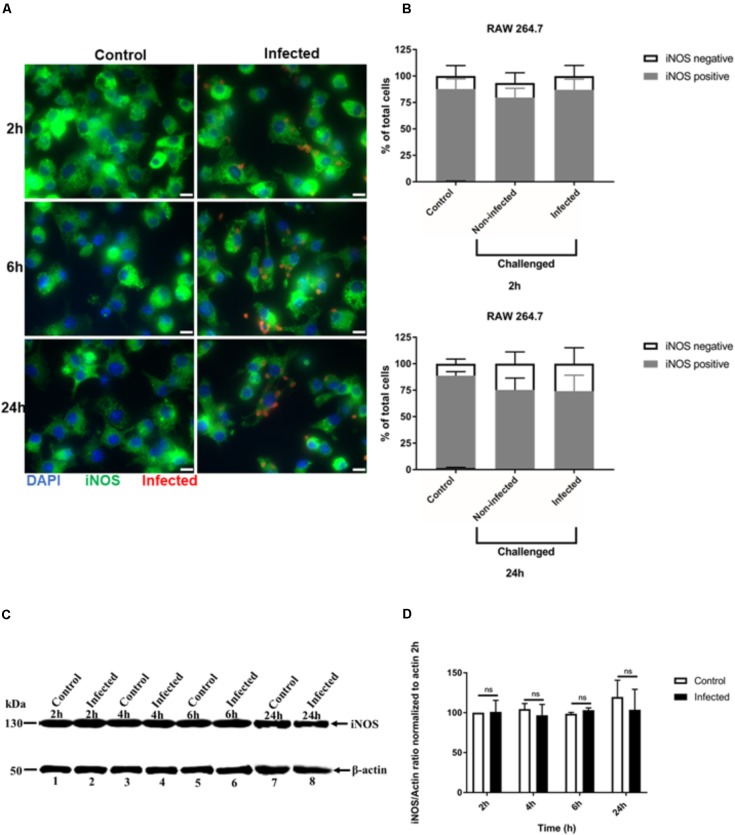
Immunofluorescence detection of iNOS in activated RAW 264.7 macrophages infected with *T. gondii*. **(A)** Detection of iNOS (green) in non-infected (Control) and in *T. gondii* (red) infected cells (DAPI - blue) at 2, 6, and 24 h post-infection. Scale bar - 10 μm. **(B)** Analysis of the proportion of iNOS positive or negative macrophages in non-infected (Control) and *T. gondii* infected cells at 2 and 24 h post-infection. Mean ± SEM (*n* = 4 experiments, each with 8 replicates). **(C)** Western blot detection of iNOS expression in non-infected (Control) and *T. gondii* - infected (Infected) cells at different time intervals post-infection. β-actin was used as loading control. **(D)** Densitometry of western bolts normalized to β-actin at 2 h post-infection. Mean ± SD (*n* = 3 experiments, each with 1 replicate), n.s (not significant).

We also analyzed iNOS expression after *T. gondii* infection of RAW 264.7 macrophages using similar IFA and Western blot analyses. The signal intensity of iNOS expression in RAW 264.7 cells was much higher, with all uninfected cells being uniformly positive (**Figure 3A** and **Supplementary Figure 2**). No difference in iNOS expression by IFA was observed between non-infected and infected RAW 264.7 macrophages at 24 h post-infection (**Figure 3A**). This finding was confirmed by analyzing the proportion of cells that were positive for iNOS. In non-infected and *T. gondii* challenged RAW 264.7 macrophage populations, most of the cells remained iNOS positive up to 24 h post-infection (**Figure 3B**). Similarly, no difference in iNOS expression between control and infected cells was observed by Western blot (**Figure 3C**) and densitometry analysis (**Figure 3D**) at different time points post-infection.

### Evaluation of *T. gondii* Virulence Factors Do Not Influence Inhibition of NO Production

**FIGURE 4:**
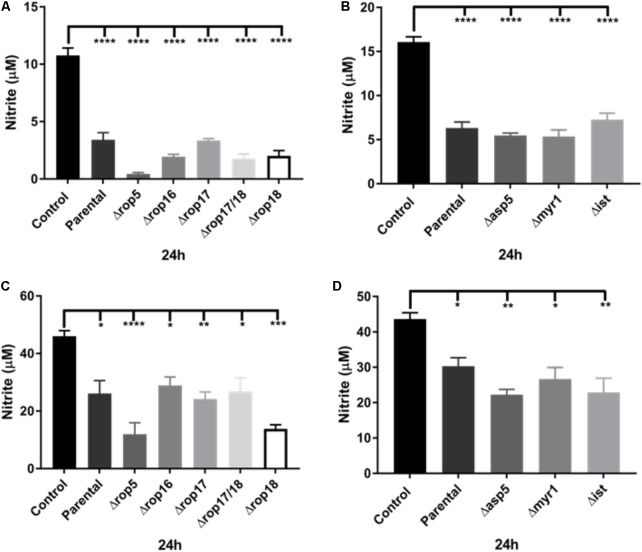
Analysis of ROP and ASP5, MYR1, and IST deletion mutants on the ability of *T. gondii* to inhibit NO production (nitrite in μM) of activated macrophages. **(A)** NO production of non-infected (Control) or activated J774-A1 cells infected with parental (RHΔku80 strain) or various ROP deletion strains of *T. gondii* at 24 h post-infection. Mean ± SEM (*n* = 3 experiments, each with 12 replicates). ^∗∗∗∗^*P* ≤ 0.0001, one-way ANOVA with Tukey post-test. **(B)** NO production of non-infected (Control) or activated J774-A1 cells infected with parental (RHΔku80 strain) or Δasp5, Δmyr1, or Δist mutant strains of *T. gondii* at 24 h post-infection. Mean ± SEM (*n* = 3 experiments, each with 12 replicates). ^∗∗∗∗^*P* ≤ 0.0001, one-way ANOVA with Tukey post-test. **(C)** NO production of non-infected (Control) or activated RAW 264.7 cells infected with parental (RHΔku80 strain) or various ROP deletion strains of *T. gondii* at 24 h post-infection. Mean ± SEM (*n* = 3 experiments, each with 12 replicates). ^∗^*P* ≤ 0.05, ^∗∗^*P* ≤ 0.01, ^∗∗∗^*P* ≤ 0.001, ^∗∗∗∗^*P* ≤ 0.0001, one-way ANOVA with Tukey post-test. **(D)** NO production of non-infected (Control) or activated RAW 264.7 cells infected with parental (RHΔku80 strain) or Δasp5, Δmyr1, and Δist mutant strains of *T. gondii* at 24 h post-infection. Mean ± SEM (*n* = 3 experiments, each with 12 replicates). ^∗^*P* ≤ 0.05, ^∗∗^*P* ≤ 0.01, one-way ANOVA with Tukey post-test.

A number of previous virulence factors have been identified in *T. gondii* including a complex of ROP kinases consisting of ROP5, ROP17, and ROP18 that participates in defense of the parasitophorous vacuole by thwarting IRGs (Hunter and Sibley, 2012). To determine whether the inhibition of NO production in infected activated J774-A1 and RAW 264.7 macrophages was dependent on the ROP kinases, we examined the inhibition of NO production in activated macrophages infected with a series of mutants. Inhibition of NO production was similar in both macrophage cell lines at 24 h infection when comparing the parent RH line to a series of ROP deletion mutants (**Figures 4A,C**). We also examined the ability of a Δrop16 mutant to alter this phenotype, since this kinase has previously been shown to activate STAT3/STAT6 and hence activate ARG1 (Butcher et al., 2011). The rop16 mutant showed a similar capacity to block NO production in activated J774-A1 and RAW 264.7 cells (**Figures 4A,C**). The various knockout parasites presented no deficiency in entry in activated J774-A1 or RAW 264.7 macrophages (**Supplementary Figure 3**).

We also tested *T. gondii* mutants in the modulator IST that inhibits STAT1 transcription (Hakimi et al., 2017). These mutants had no effect on the NO inhibition phenotype (**Figures 4B,D**). Recently, the major role of the Golgi-associated protein, ASP5, and the parasitophorous vacuole (PV) associated protein, MYR1, in the cleavage and export of some dense granule effector proteins across the vacuole membrane into the host cell has been demonstrated (Curt-Varesano et al., 2016; Franco et al., 2016). Therefore we examined the ability of mutants in these effectors to block the production of NO in activated macrophages. After infection, all knockout parasites were able to inhibit NO production of activated J774-A1 (**Figure 4B**) and RAW 264.7 macrophages (**Figure 4D**) similar to the parental parasite. The various knockout parasites presented no deficiency in entry in activated J774-A1 or RAW 264.7 macrophages (**Supplementary Figure 3**).

## Discussion

*Toxoplasma gondii* has many evasion mechanisms including the capacity to inhibit NO production of infected activated macrophages (Dobbin et al., 2002; Seabra et al., 2002, 2004; Luder et al., 2003; Padrao Jda et al., 2014). NO production inhibition, iNOS expression and the role of some *T. gondii* effectors were studied in parallel using two macrophage cell lines. Infection of both cell lines caused inhibition of NO production. However, only in J774-A1 macrophages was NO inhibition detected at early stages of infection (i.e., 2 h post-infection), while inhibition was seen starting at 6 h post-infection in both lines. Addition of extra L-arginine substrate to both macrophage lines did not change NO production inhibition, indicating that this result is not due to substrate limitation. Interestingly, reduction of iNOS expression after infection was only detected in J774-A1 cells, with RAW 264.7 presenting the same levels as non-infected cells. Finally, knockout parasites in known effectors were able to inhibit NO production similar to the parental strain. Our findings suggest that the inhibition of NO production of activated macrophages infected by *T. gondii* is a general phenomenon, but iNOS suppression varies depending on the macrophage cell line. In addition, it is likely that a novel parasite effector is responsible for this evasion mechanism.

*Toxoplasma gondii* infection causes ARG1 expression that competes with iNOS for L-arginine (El Kasmi et al., 2008; Butcher et al., 2011). However, extra L-arginine did not reverse NO production inhibition in either infected cell lines, indicating that the reduction in NO production is not due to substrate limitation. Following addition of L-arginine, J774-A1 produced the same amount of NO while RAW 264.7 macrophages responded to this addition by producing more NO. This finding is consistent with the differences in iNOS expression, and provides a further distinction in phenotypes between these two lines (Heming et al., 2001; Lindmark et al., 2004; El Aamri et al., 2015).

Previous report showed that NO production inhibition of *T. gondii* infected activated mice peritoneal macrophages was related to iNOS degradation (Seabra et al., 2002, 2004), which involves the proteasome in J774-A1 macrophages (Padrao Jda et al., 2014). Down modulation of iNOS expression was verified in infected J774-A1 but this was not observed in RAW 264.7 macrophages. These results suggest that the strategy adopted by *T. gondii* to inhibit NO production may be specific to the host cell, due to the intrinsic characteristics and origin of each macrophage cell line. The J774-A1 line was originally established from reticulum cell sarcoma (Hirst et al., 1971) while the RAW 264.7 line from a tumor induced by Abelson murine leukemia virus (Ralph and Nakoinz, 1977) both in BALB/c mice. Furthermore, exposure of both cell lines to *Streptococcus iniae* induces a higher respiratory burst response in RAW 264.7 than in J774-A1 macrophage (El Aamri et al., 2015). Moreover, RAW 264.7 produces 30-fold higher TNF-α mRNA than J774-A1 after LPS stimulation (Heming et al., 2001). These differences help to explain the higher expression of iNOS in RAW 264.7 compared to J774-A1 macrophages. In addition, a gene expression profile study (Lindmark et al., 2004) shows that J774-A1 is closer to peritoneal mice macrophages than RAW 264.7, despite the fact that both cell lines were derived from transformed cells obtained from ascites (Ralph and Nakoinz, 1977). Our findings reveal that *T. gondii* infection can down-regulate NO production in these different macrophage cell lines, albeit by different mechanisms.

During host cell infection, *T. gondii* secrets the content of rhoptries and dense granules ensuring the establishment of infection and hijacking host cell-autonomous immunity (Sibley, 2011). ROP and GRA proteins form complexes that protect *T. gondii* PV by avoiding recruitment of IRGs (Hunter and Sibley, 2012). GRA proteins are also exported across the PV altering important host functions (Hakimi et al., 2017). TgIST is a GRA effector that represses STAT1 transcription blocking gene expression induced by IFN-γ (Gay et al., 2016; Olias et al., 2016). In addition, the export and traffic of GRAs across the PV and into the host cell are dependent of ASP5 (Coffey et al., 2015; Curt-Varesano et al., 2016) and MYR1 (Franco et al., 2016). However, these virulence factors have not previously been evaluated for their ability to modulate other important host microbicidal systems such as NO production. Thus, knockout parasites were used to investigate whether some rhoptry proteins (ROP5, ROP16, ROP17, ROP18). The parasite ROP16 kinase has been implicated in modulating NO production in microglial cells and astrocytes (Butcher et al., 2011). However, the knockout in ROP16 did not affect the down modulation of NO production in either of the macrophage cells lines studied here. Additionally, the ROP5-ROP17-ROP18 complex, which has been implicated in blocking IRG-mediated clearance, did not affect the down modulation of NO production in *T. gondii* – infected cells. We also explored the roles of the GRA TgIST, or components of the PV membrane translocation system (ASP5, MYR1) for involvement in NO production control in infected macrophages. These GRA knockout parasites were able to inhibit NO production similar to the parental strain in J774-A1 and RAW 264.7 macrophages. These results indicate that the parasite effector that down modulates NO production is independent of TgIST, including other modulators that depend on the trafficking pathway based on ASP5 and MYR1.

Overall, our findings reveal that the strategy adopted by *T. gondii* to inhibit NO production in activated macrophages is independent of previously characterized virulence factors, such as the ROP5-ROP17-ROP18 complex, ROP16, GRA effectors, and the ASP5 and MYR1 export pathway. Thus, a possibly new parasite effector is involved in NO production inhibition in these cells. The mechanism causing NO production inhibition of macrophages infected by *T. gondii* varies depending on the host cell background: it involves reduction of iNOS expression in J774-A1 and iNOS impairment in RAW 264.7. This may be relevant to *in vivo* infections where *T. gondii* infects and needs to cope with distinct macrophage populations.

## Author Contributions

GC performed the experiments. ZW produced the ROP16 knockout. GC, LS, and RD wrote the manuscript. LS and RD designed the experiments and revised the manuscript critically. All authors read and approved the final manuscript.

## Conflict of Interest Statement

The authors declare that the research was conducted in the absence of any commercial or financial relationships that could be construed as a potential conflict of interest.

## Abbreviations

ASP5: aspartyl protease 5
GRA: dense granule proteins
iNOS: inducible nitric oxide synthase
IRGs: immunity related GTPases
MYR1: myc regulation 1
NO: nitric oxide
ROP: rhoptry proteins
TgIST: *Toxoplasma gondii* inhibitor of STAT1-dependent transcription
