# Supplementary material for: Inhibition of Nitric Oxide Production in Activated Macrophages Caused by Toxoplasma gondii Infection Occurs by Distinct Mechanisms in Different Mouse Macrophage Cell Lines
Source: Front Microbiol. 2018 Aug 20;9:1936. doi: 10.3389/fmicb.2018.01936 (PMC6109688; doi:10.3389/fmicb.2018.01936)
Supplement: Supplementary file 1 [file Data_Sheet_1.pdf]

Inhibition of Nitric Oxide Production in Activated Macrophages Caused by *Toxoplasma gondii* Infection Occurs by Distinct Mechanisms in Different Cell Lines

Gabriel R. A. Cabral, Zi Teng Wang, L. David Sibley *, and Renato A. DaMatta*

*** Correspondence:** Dr. L. David Sibley: sibley@wustl.edu; Dr. Renato Augusto DaMatta: renato@uenf.br

Supplementary Figures

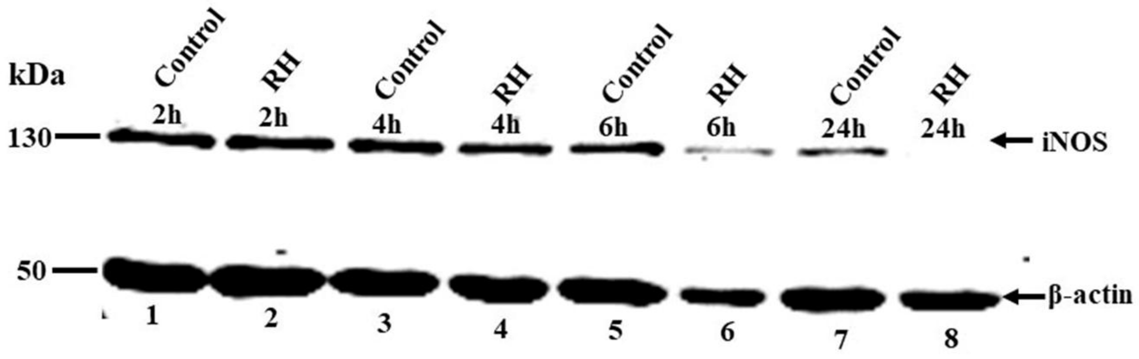

Supplemental figure 1. Western blot detection of iNOS expression in non-infected (Control) and *T. gondii* infected (RH) activated J774-A1 macrophages. β-actin was used as loading control.

A)

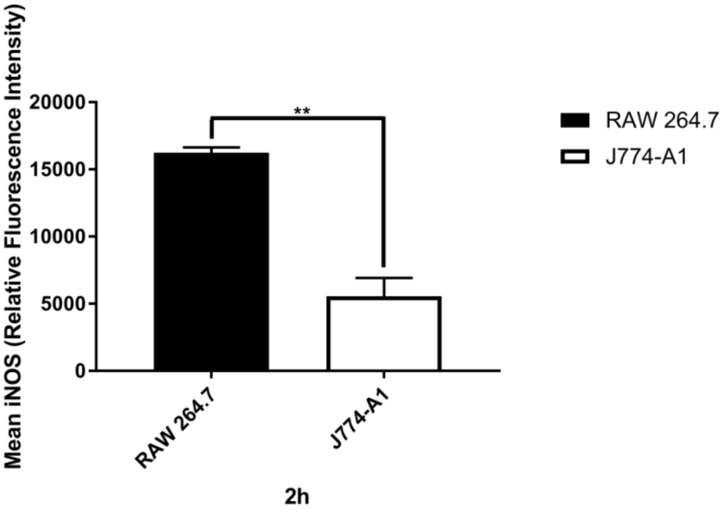

B)

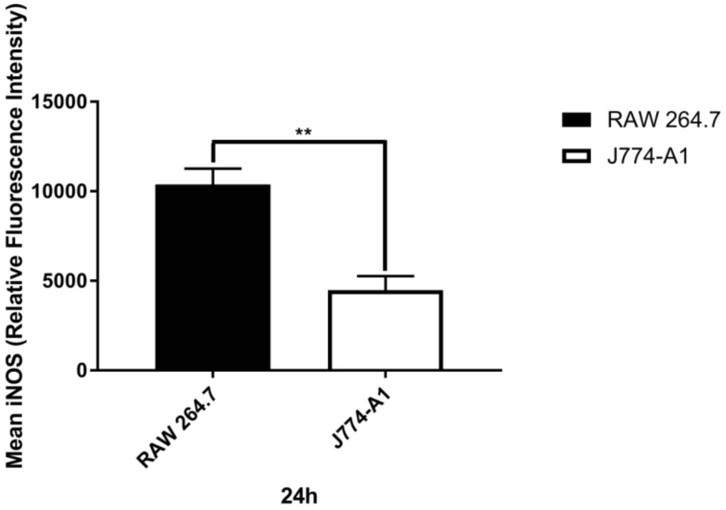

Supplemental figure 2. Relative expression of iNOS in activated RAW 264.7 and J774-A1 macrophages. (A) iNOS expression in control RAW 264.7 and J774-A1 macrophages at 2 h and (B) 24 h post-infection. Mean ± SEM (n = 4 experiments, each with 8 replicates). ***P*≤0.01, unpaired Student t test.

A) J774-A1

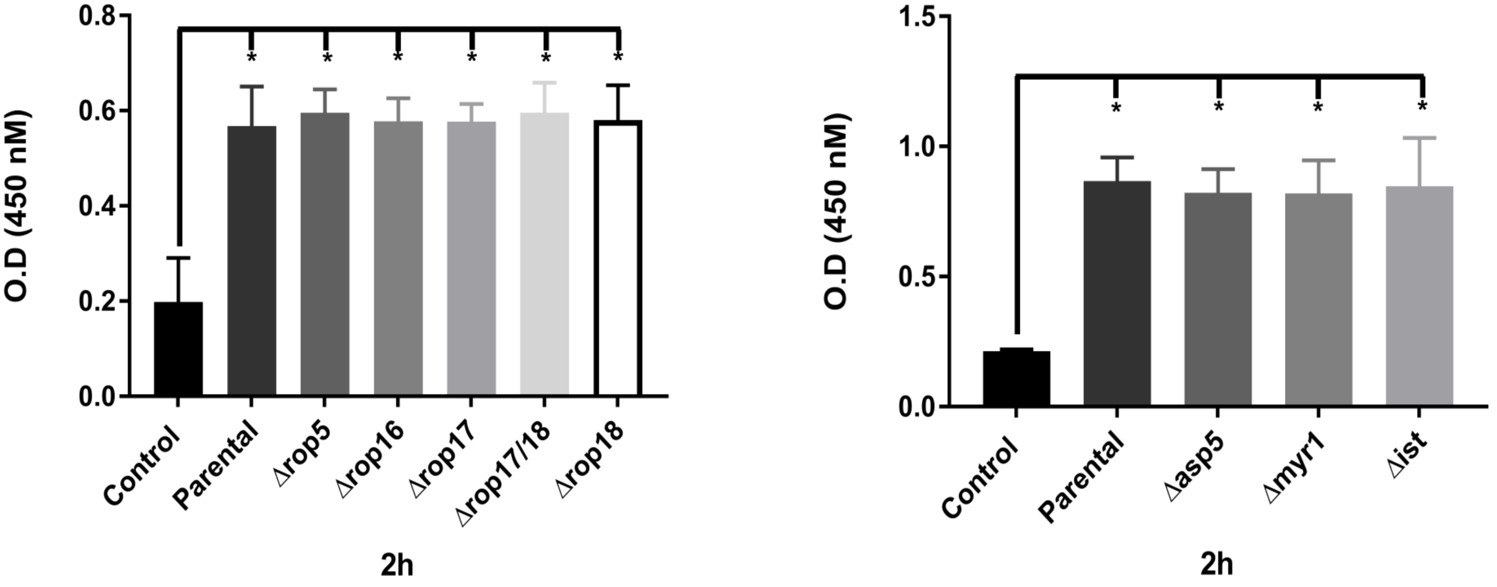

B) RAW 264.7

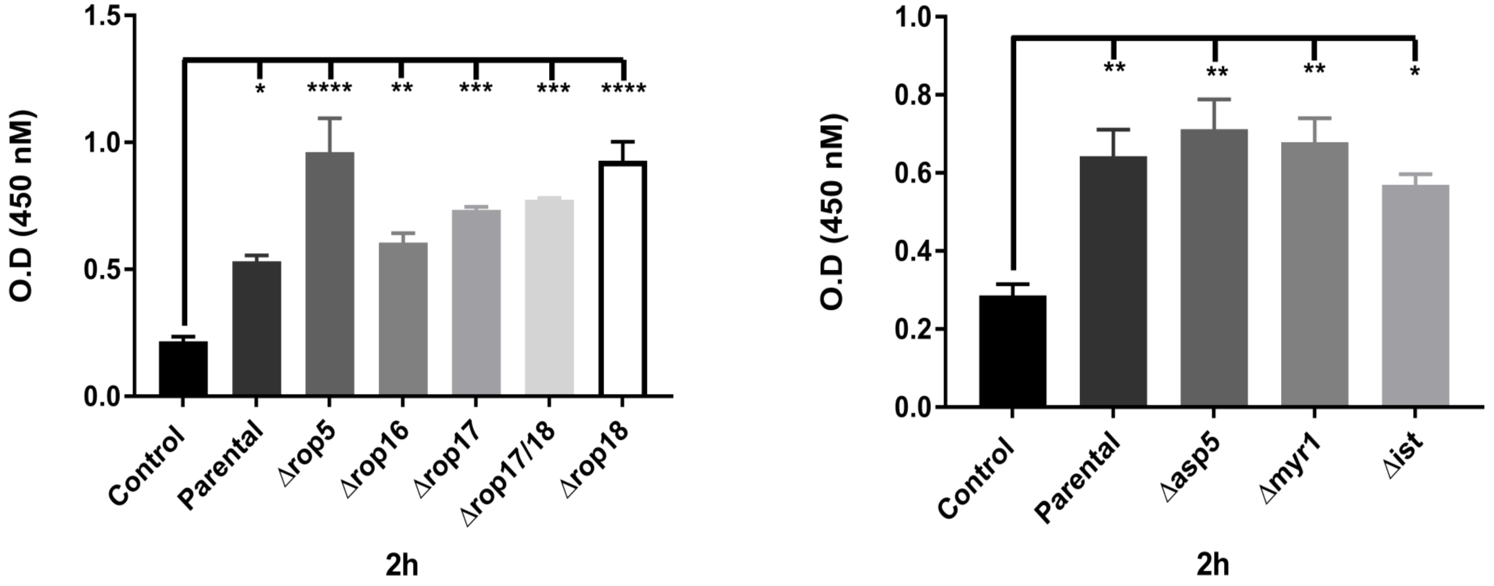

Supplemental figure 3. Analysis of entry by parental and knockout *T. gondii* in activated J774-A1 and RAW 264.7 macrophages at 2 h post-infection. (A) Infection of activated J774-A1 macrophages by parental (RHΔku80) and mutant strains of *T. gondii*. Mean ± SEM (n = 3 experiments, each with 12 replicates). (B) Infection of activated RAW 264.7 macrophages by parental (RHΔku80) and mutant strains of *T. gondii*. Mean ± SEM (n= 3 experiments, each with 12 replicates). *$P \leq 0.05$, **$P \leq 0.01$, ***$P \leq 0.001$, ****$P \leq 0.0001$, one-way ANOVA with Tukey post-test.
